# Supplementary material for: Glycoprotein 5-Derived Peptides Induce a Protective T-Cell Response in Swine against the Porcine Reproductive and Respiratory Syndrome Virus
Source: Viruses. 2023 Dec 21;16(1):14. doi: 10.3390/v16010014 (PMC10819526; doi:10.3390/v16010014)

SUPPLEMENTARY MATERIAL

Figure S1. Immunization and blood sampling schedule

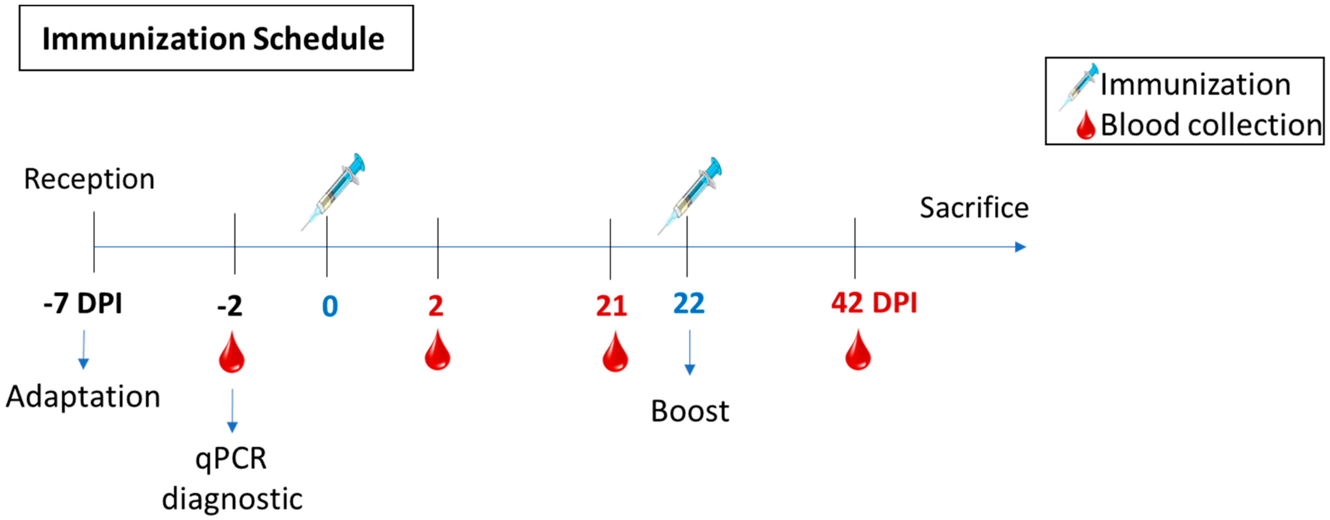

Figure S2. Immunophenotyping CD3+/CD8+and CD8+/CD44+ cells population from lymphocytes.

Selection of single cells from PBMC

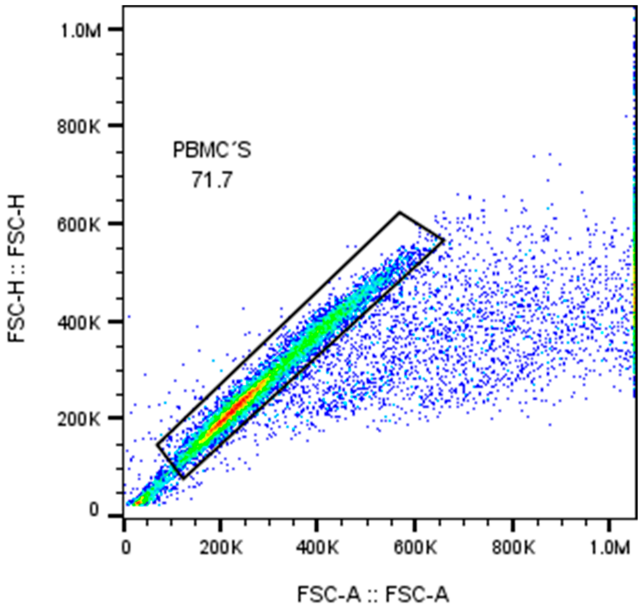

Lymphocyte populations from singlets were separated based on their size and complexity.

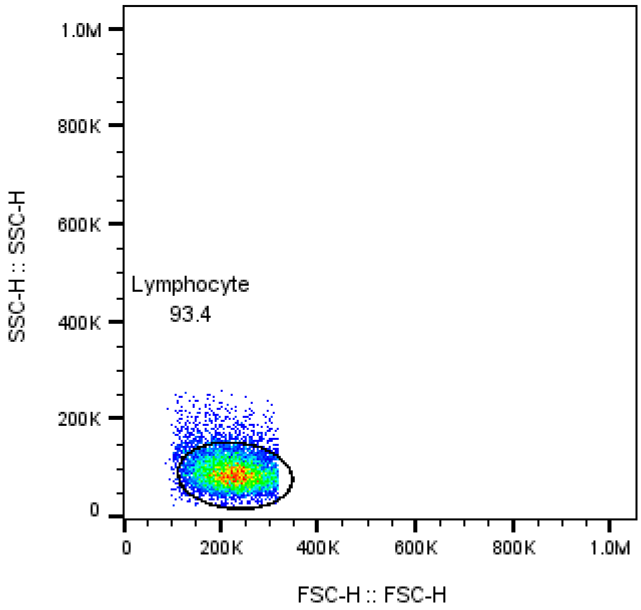

CD3+/CD8+ cells from the total lymphocyte population.

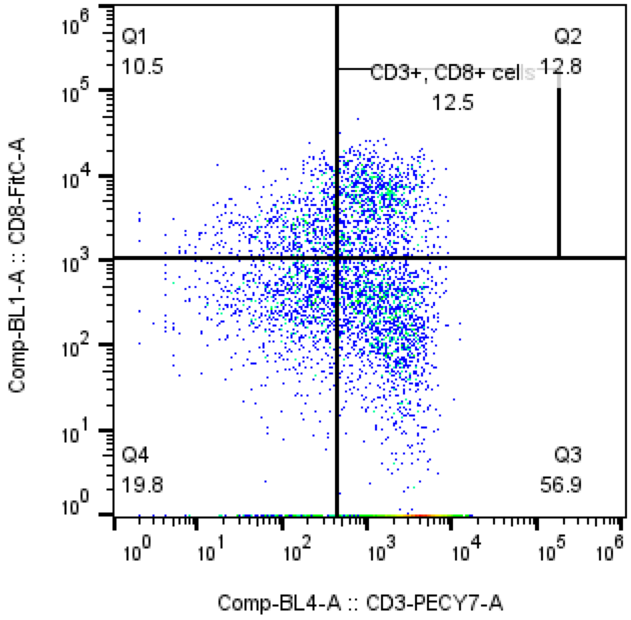

CD8+/CD44+ cells from the total lymphocyte population.

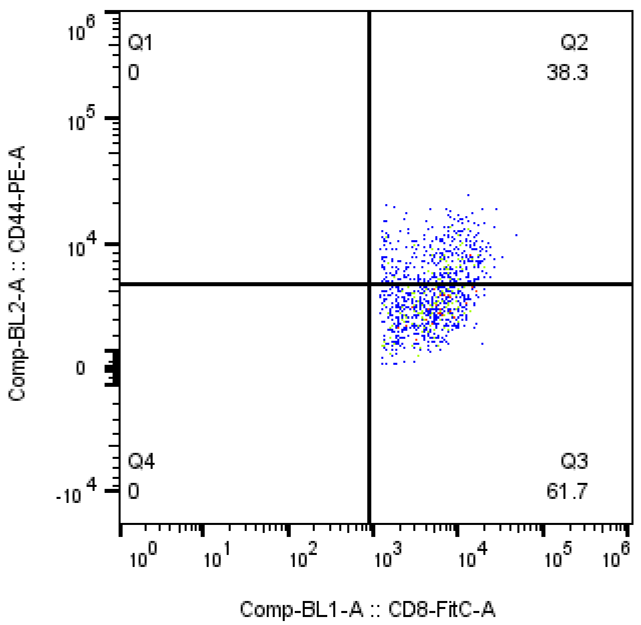

Figure S3**.** Standard curve for PRRSV quantification.

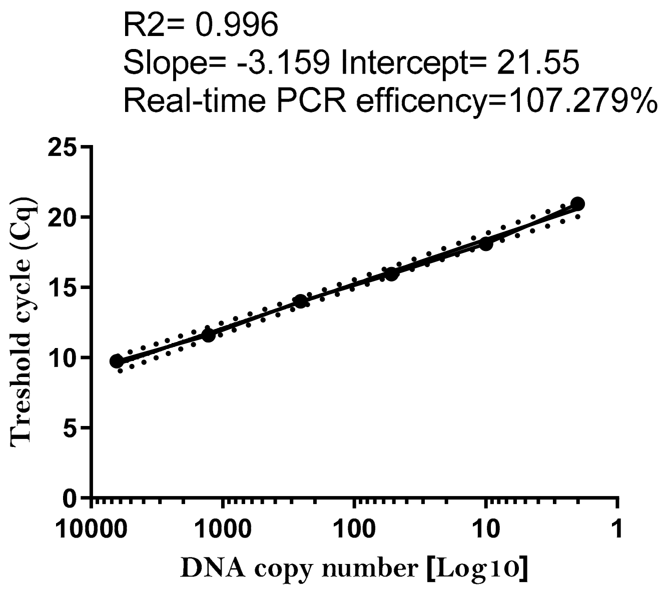

Standard curve for the quantification of viral copy number using qPCR.

We used six-fold dilutions ranging from 6.4 x 10^9 to 2.0 x 10^6 copies, based on Log10 per µl.

These dilutions were plotted with the dilution factor on the X-axis and the quantification cycle (Cq) values on the Y-axis.

Figure S4. Viability of the lymphocytes after cryopreservation assessed using MTT assay.

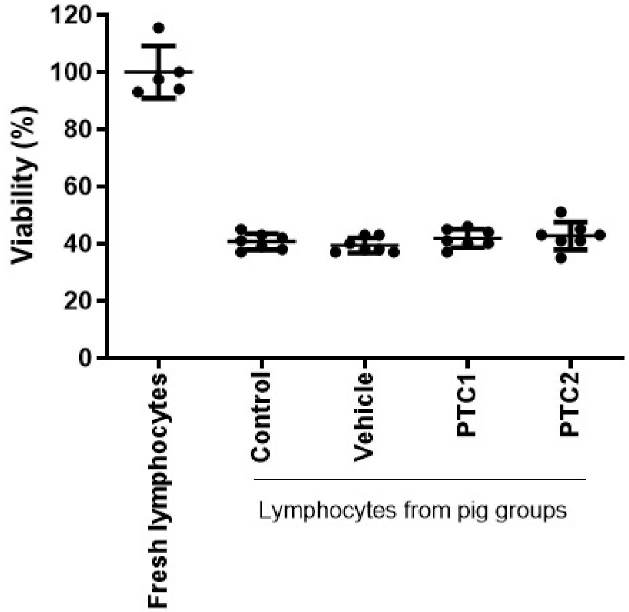

Figure S5. Graphical Representation of qPCR Software Results for the Detection of PRRSV in Experimental Pig Samples.

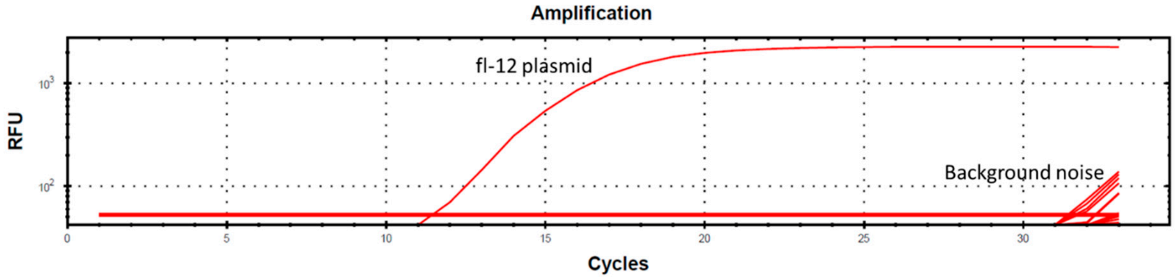

Supplement: Supplementary file 1 [file viruses-16-00014-s001.zip › viruses-2761396-supplementary.pdf]
